# Contrast Extravasation Mimicking Subarachnoid Hemorrhage After Cardiac Catheterization

**DOI:** 10.7759/cureus.9212

**Published:** 2020-07-15

**Authors:** Syed A Huda, Moeed Ahmed, Parth J Sampat, Bashar Ibeche, Bashar Sharma

**Affiliations:** 1 Internal Medicine, State University of New York (SUNY) Upstate Medical University, Syracuse, USA; 2 Internal Medicine, Creighton University School of Medicine, Nebraska, USA

**Keywords:** contrast extravasation, subarachnoid hemorrhage, coronary angiography, contrast toxicity

## Abstract

Neurological complications after coronary angiography are rare but associated with significant mortality and morbidity. These include ischemic and hemorrhagic strokes, and transient ischemic attacks. Rarely, contrast media can cross the blood brain barrier causing transient neurological symptoms including confusion and seizures. On imaging, it can mimic a subarachnoid hemorrhage (SAH). Blood can be differentiated from contrast media using MRI. We present a patient who developed confusion after undergoing cardiac angiography and the initial CT of the brain showed SAH. However, MRI of the brain did not reveal any hemorrhage indicating contrast staining.

## Introduction

Coronary angiography is the gold standard for diagnosis and treatment of coronary artery disease. Due to technological advancements, the rate of major cardiac and cerebrovascular events (MACCEs) in patients undergoing percutaneous coronary intervention is only 2.5% [1]. Cerebrovascular events (CVE) including ischemic and hemorrhagic stroke and transient ischemic attacks are rare after cardiac catheterization but can lead to significant morbidity [2]. With the development of more potent anti-platelet medications, there is also an increased risk of bleeding [3]. The incidence of major bleeding after coronary angiography is 2.7% and in patients with acute coronary syndrome undergoing percutaneous coronary intervention, the bleeding rate is about 18.1% [4]. In some cases, there is extravasation of contrast due to increased permeability of the blood brain barrier, mimicking an acute subarachnoid hemorrhage (SAH) on CT of the head [5]. We present a case of contrast leakage into the subarachnoid space imitating SAH on CT scan.

## Case presentation

A 59-year-old man presented to the ED due to decreased oral intake for a few days. His medical problems included hypertension, diabetes mellitus type II, hereditary hemochromatosis, hereditary elliptocytosis, and myelodysplastic syndrome. On further questioning, the patient mentioned that he had an episode of midsternal chest pressure earlier in the day and it lasted for a couple of hours. It was associated with shortness of breath, but he denied any palpitation or diaphoresis. The pain did not recur, and he was pain free in the ED.

On arrival, his blood pressure was 139/84 mmHg, heart rate was 72 beats per minute, temperature was 97.6°F, and respiratory rate of 18 per minute with an oxygen saturation of 97% on room air. Cardiac exam revealed regular rate and rhythm with normal S1 and S2. His lungs were clear to auscultation bilaterally. He had no cyanosis, clubbing, or lower extremity swelling.

**Figure 1:**
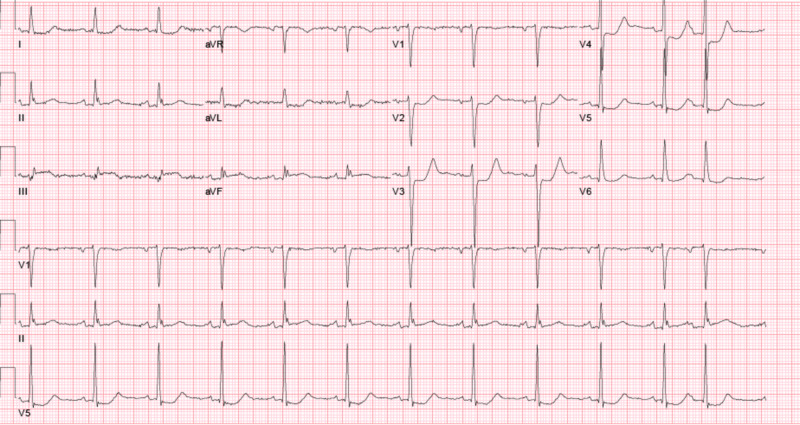
EKG shows ST elevation in Lead III and aVF and reciprocal ST depressions in lead V2, V3, and V4. EKG, electrocardiogram

Initial labs showed a sodium level of 134 mEq/L (135-145 mEq/L), potassium 4.2 mEq/L (3.8-5.1 mEq/L), blood urea nitrogen 21 mg/dL (9-26 mg/dL), serum creatinine 1.1 mg/dL (0.7-1.4 mg/dL), white blood cell count 7,700 per mm3 (4.4-10.7 K/mm3), hemoglobin 10.6 g/dL (13.5-17 g/dl), troponin I 12.30 ng/dL (0-0.05 ng/dL), creatine kinase-MB (CK-MB) 14.7 ng/mL (0.0-5.0 ng/ml), and creatine phosphokinase (CPK) 140 units/L (21-232 units/L). Electrocardiogram (EKG) showed ST segment elevations in the inferior leads with reciprocal ST depressions in the anterior leads (Figure 1).

 

**Figure 2:**
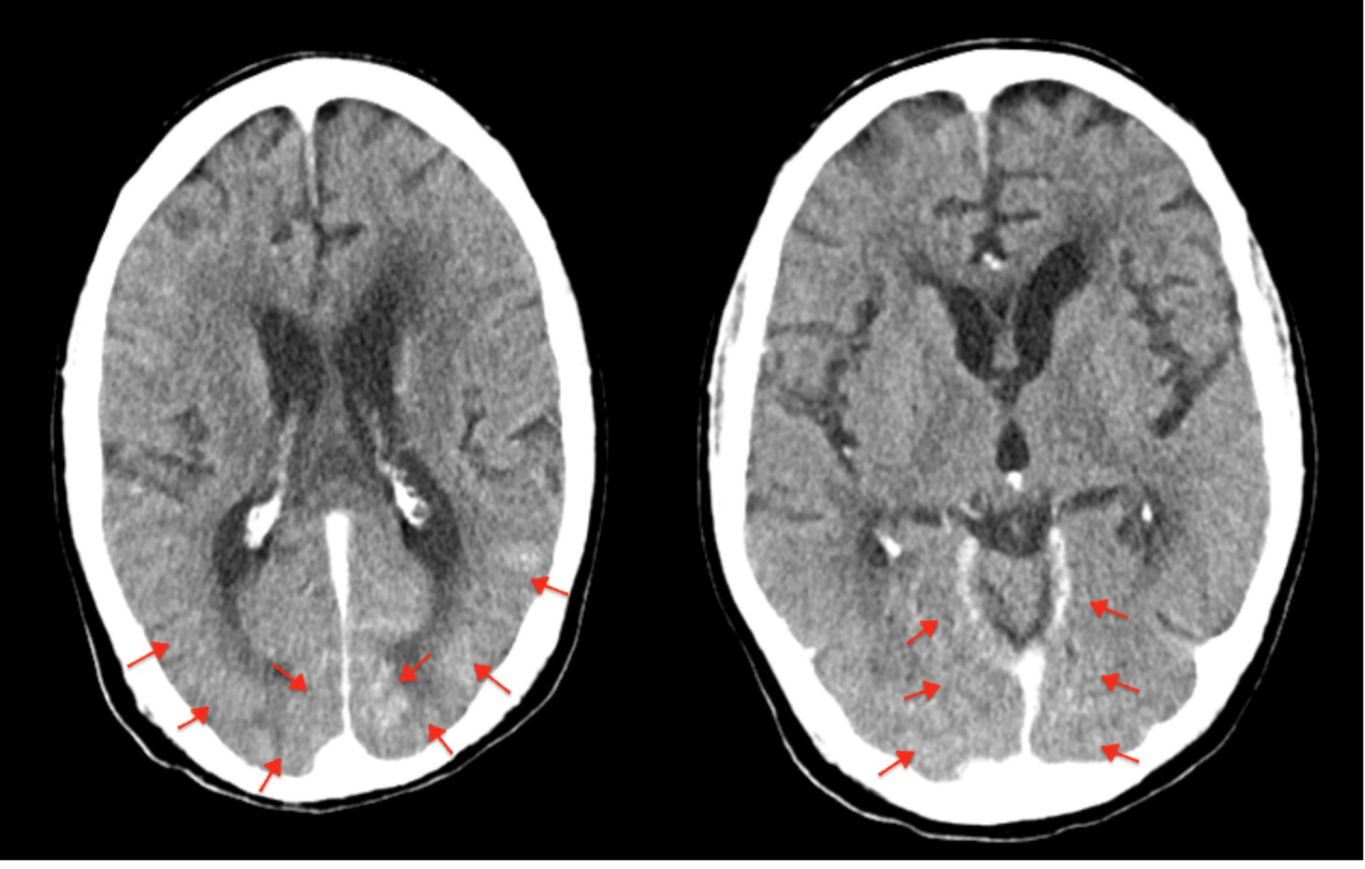
Contrast staining mimicking subarachnoid blood products in the posterior left and right parietal lobes.

**Figure 3:**
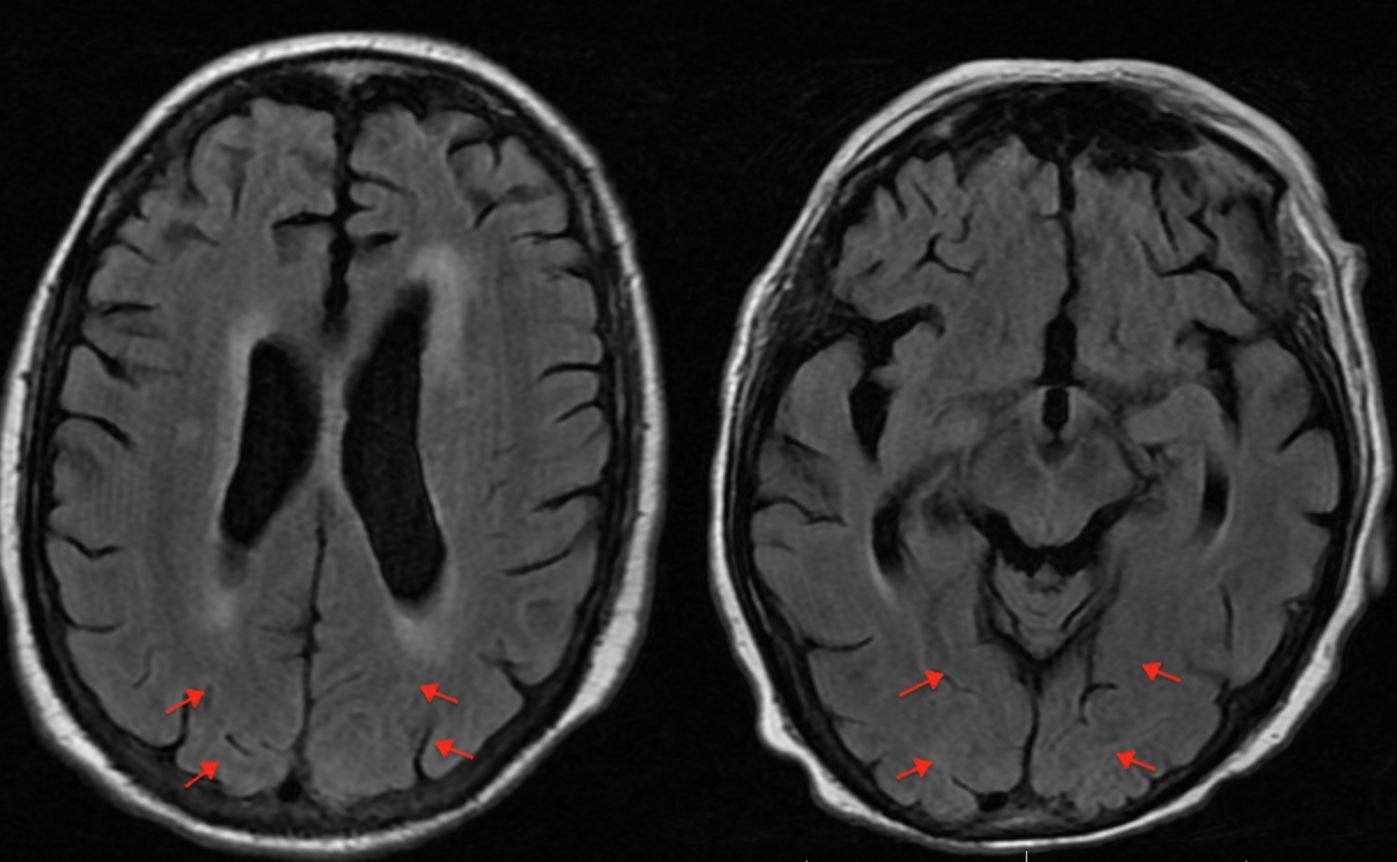
No SAH seen on MRI brain. SAH, subarachnoid hemorrhage

The patient was taken for emergent cardiac catheterization and it revealed a complete occlusion of the right coronary artery (RCA). He underwent a successful percutaneous intervention with a drug eluting stent to the RCA. After the procedure, the primary team noticed that the patient was confused and lethargic that was concerning for an acute stroke. Neurological exam, however, did not reveal any focal deficit. A CT head done three hours post procedure showed subarachnoid blood products involving the left and right parietal lobes as well as the occipital lobes with local sulcal effacement and gyral edema (Figure 2). These findings were consistent with acute SAH. His antiplatelets were held and he was transferred to the ICU for strict blood pressure control. Due to these concerning findings, an MRI brain was performed 12 hours later which did not show SAH but did reveal multiple acute infarcts in the bilateral frontal lobes (Figure 3). His antiplatelets were restarted. Stroke work up included an echocardiogram that did not reveal any patent foramen ovale or left atrial appendage thrombus and imaging of the carotid arteries that did not show any significant stenosis. He was diagnosed with an embolic stroke of unknown origin and sent home with a loop recorder.

## Discussion

Subarachnoid hemorrhage is a life-threatening complication of coronary angiography. However, leakage of contrast media into the subarachnoid space, also known as contrast media neurotoxicity, can mimic SAH on imaging. It results from disruption of the blood brain barrier. Contrast media neurotoxicity is usually short lived with rapid reversal [4]. It is rare but occurs more frequently with the use of ionic contrast media as compared to nonionic contrast media [6] . Other risk factors include renal insufficiency, male sex, advanced age, hypertension, impaired kidney function, injection of large volumes of contrast media, and underlying brain disturbances [7].

On CT scans, the radiodensity of blood normally range from 0 to 60 Hounsfield units (HU), with 100% blood by volume having an attenuation of 66 HU. On the other hand, maximal density of contrast medium is more than 90 HU. Blood can be differentiated from contrast medium by using MRI. On MRI, T1 relaxation times for blood/cerebrospinal fluid (CSF) mixture decreases as the blood concentration increases, ranging from 2200 to 500 milliseconds (ms) for 100% CSF and 100% blood respectively, while T2 findings for CSF/blood mixtures are similar to T1 relaxation times [8].

In our patient the initial CT head was concerning for SAH for which his antiplatelets were held, which increased the patient's risk of developing a stent thrombosis. The MRI of the brain done the next day did show multiple ischemic strokes but no hemorrhage allowing safe resumption of dual antiplatelet therapy. Though it is unfortunate that our patient suffered from an ischemic stroke, having a SAH would have placed him at a significantly increased mortality and morbidity from both a neurological and cardiac standpoint.

## Conclusions

In conclusion, patients who have CT findings suggestive of hemorrhage after coronary angiography should have prompt MRI to rule out contrast extravasation. In centers that lack the facility of an MRI, repeat CT imaging should be performed as hyperdensity seen by contrast extravasation improves rapidly. Erroneous diagnosis of SAH can lead to increased instances of in-stent thrombosis due to discontinuation of antiplatelet therapy.
